# Controlateral Symmetrisation in SRM for Breast Cancer: Now or Then? Immediate versus Delayed Symmetrisation in a Two-Stage Breast Reconstruction

**DOI:** 10.3390/curroncol29120737

**Published:** 2022-11-30

**Authors:** Donato Casella, Daniele Fusario, Dario Cassetti, Anna Lisa Pesce, Alessandro De Luca, Maristella Guerra, Roberto Cuomo, Diego Ribuffo, Alessandro Neri, Marco Marcasciano

**Affiliations:** 1 Department of Medicine, Surgery and Neurosciences, Unit of Breast Cancer Surgery, University of Siena, 53100 Siena, Italy; 2 Department of Medicine, Surgery and Neurosciences, Unit of General Surgery and Surgical Oncology, University of Siena, 53100 Siena, Italy; 3 Unit of General Surgery, USL Toscana Sud-Est, Valdarno Hospital Santa Maria alla Gruccia, 52025 Arezzo, Italy; 4 Department of Surgery, Sapienza Università di Roma, 00185 Rome, Italy; 5 Unit of Plastica Surgery, Polo Ospedaliero Santo Spirito ASL/RME, 00193 Rome, Italy; 6 Department of Medicine, Surgery and Neurosciences, Unit of Plastic and Reconstructive Surgery, University of Siena, 53100 Siena, Italy; 7 Department of Plastic Reconstructive and Aesthetic Surgery, Sapienza Università di Roma, 00185 Rome, Italy; 8 Unit of Breast Surgery, USL Toscana Sud-Est, San Donato Hospital, 52100 Arezzo, Italy; 9 Unit of Plastic and Reconstructive Surgery, University of Catanzaro “Magna Graecia”, 88100 Catanzaro, Italy

**Keywords:** breast reconstruction, skin-reducing mastectomy, implant-based breast reconstruction, subcutaneous implant positioning, controlateral breast symmetrisation

## Abstract

*Introduction:* The timing of contralateral symmetrisation in patients with large and ptotic breasts undergoing a unilateral skin-reducing mastectomy (SRM) is one of the most debated topics in the reconstructive field. There is no evidence to support the advantage of immediate or delayed symmetrisation to help surgeons with this decision. The aim of this study was to investigate the clinical and aesthetic outcomes of immediate symmetrisation. *Methods*: A randomised observational study was conducted on patients who underwent an SRM for unilateral breast cancer. Based on a simple randomisation list, patients were divided into two groups: a delayed symmetrisation group versus an immediate symmetrisation group. The postoperative complications, BREAST-Q outcomes and reoperations were compared. *Results:* Out of a total of 84 patients undergoing an SRM between January 2018 and January 2021, 42 patients underwent immediate symmetrisation and 42 patients had delayed symmetrisation. Three implant losses (7.2%) were observed and we reported three wound dehiscences; one of these was in a contralateral breast reconstruction in the immediate symmetrisation group. The BREAST-Q patient-reported outcome measures recorded better aesthetic outcomes and a high patient satisfaction for the immediate symmetrisation group. *Conclusions*: Simultaneous controlateral symmetrisation is a good alternative to achieve better satisfaction and quality of life for patients; from a surgical point of view, it does not excessively impact on the second time of reconstruction.

## 1. Introduction

In 1991, Toth et al. [1] first described a skin-sparing mastectomy (SSM). By preserving the breast envelope and inframammary fold, a much more satisfactory cosmetic outcome could be achieved during a reconstruction.

Rice and Stickler in 1951 [2] described an “adeno-mammectomy” for benign diseases and Freeman in 1962 [3] presented the term “subcutaneous mastectomy”: these are the first descriptions of a nipple-sparing mastectomy (NSM). An NSM is similar to an SSM for the dissection of skin flaps, but considers the NAC.

In patients with large and ptotic breasts that are higher than the second degree according to the Regnault classification [4], it is difficult to approach a mastectomy because an excellent satisfactory aesthetic outcome is hard to obtain [5].

Carlson et al. [6] in 1997 described four types of incision that could be used for an SSM; in particular, the Wise pattern is used for those patients with medium-sized or large ptotic breasts. Therefore, these authors first described a technique that combined a skin-sparing mastectomy with a simultaneous reduction of the breast envelope. For many years, this was not universally known; thus, in 2006, Nava et al. reproposed and renamed this technique the skin-reducing mastectomy (SRM) [7].

Although the results were reassuring, patients with macromastia and ptotic breasts remained a stimulating group to treat; the timing of contralateral symmetrisation remains one of the most debated topics in the breast reconstruction field [8,9,10].

Nowadays, breast surgeons regularly try to perform a symmetrical and aesthetically pleasing breast reconstruction to achieve a better outcome.

Despite the pros and cons of immediate versus delayed symmetrisation being well-documented, the ideal moment for performing a contralateral surgical procedure remains debated [11].

Currently, immediate symmetrisation is a questioned procedure. On one hand, a few surgeons prefer delayed symmetrisation to reduce the operating times and blood loss, thus potentially decreasing the morbidities. Additionally, important fat necrosis or partial flap losses may impose a change in the plan for reconstructed and contralateral breasts. On the other hand, several surgeons prefer immediate symmetrisation in order to give the patient immediate psychological wellness and increase their quality of life by the immediate reduction of asymmetry and, furthermore, to reduce the number of postoperative expansions needed to the reach the final volume and to avoid another operation. 

The purpose of this study was to investigate the clinical and aesthetic outcomes of immediate symmetrisation and to suggest our indication in an attempt to help surgeons with this operative decision.

## 2. Materials and Methods

This was a randomised observational study conducted on a population of patients with a diagnosis of unilateral breast cancer who underwent an SRM with a prepectoral tissue expander (Mentor CPX4, Mentor Worldwide LLC, Irvine, CA, USA) reconstruction implanted with specific covering devices (TiLoop^®^ Bra, PFM medical, Cologne, Germany) followed by a substitution with a silicon-based implant at a later stage [12,13]. The enrolment started in January 2018 and ended in January 2021 at the Unit of Oncological Breast Surgery, University of Siena.

All women had a confirmed breast cancer diagnosis, were aged 18 years or older, met the criteria suggested by Nava et al. [7] for an SRM (patients with medium to large breasts with breast ptosis and at least grade II from the Regnault classification) and had a Pre-BRA score [14] from five to eight, indicating the implant of a prepectoral tissue expander and a subcutaneous definitive prosthesis from a second-time surgery. 

The exclusion criteria were clinical evidence of axillary metastases or skin or chest wall tumour involvement, a body mass index greater than 35 kg/m^2^, pregnancy, active smokers, connective tissue disease, diabetes, previous thoracic radiotherapy and previous breast surgery.

All data were collected upon informed consent acceptance and when the patients enrolled had accepted contralateral symmetrisation. We then divided the patients into two groups: one with delayed symmetrisation and one with immediate symmetrisation, based on a simple randomisation list using a dedicated computer program. 

The patient data (including the age, body mass index and treatment characteristics as well as the indication for surgery, including the type of cancer, axillary surgery and locoregional or systemic recurrence, surgical complications and aesthetic outcomes) were collected from our specifically designed database.

The study was accomplished according to the ethical standards of the Declaration of Helsinki. Ethics approval was not required because the different timings of contralateral symmetrisation did not require any modifications to the standard therapeutic protocols.

**Figure 1:**
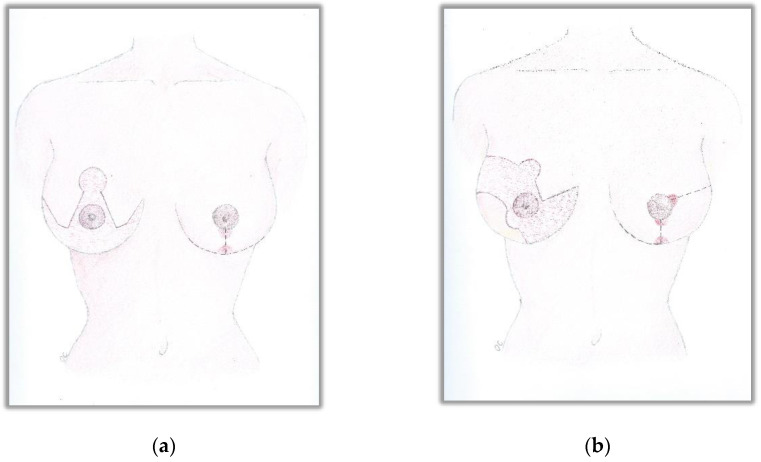
(**a**) Wise pattern incision for SRM; (**b**) modified Wise pattern incision for SRM used to remove the skin overlying or infiltrated by the tumour in the lateral quadrants of the breast.

All SRMs were conducted by a Wise pattern incision or a modified Wise pattern incision used to remove the skin overlying or infiltrated by the tumour in the lateral quadrants of the breast, as shown in Figure 1; in all cases, the nipple–areola complex (NAC) was removed at the beginning of the surgical procedure and reimplanted with the free-nipple graft (FNG) technique at the end of the surgical operation.

For the symmetrisation procedure, the patients underwent a reduction mammoplasty performed by a Wise pattern incision. 

All the patients underwent an intraoperative sentinel lymph node (SLN) examination by a one-step nucleic acid amplification (OSNA) assay (Sysmex, Kobe, Japan) [15].

A health-related quality of life (HRQOL) evaluation was conducted using the preoperative and postoperative BREAST-Q modules. It has been largely corroborated for research in breast reconstruction and is routinely used at our institutions [16,17].

After a consultation with the oncology and plastic surgeon, the enrolled patients received the preoperative questionnaire 1 month before surgery. The BREAST-Q postoperative modules were administered 1 year after the breast reconstruction. All aspects of the BREAST-Q reconstructive modules (satisfaction with the breasts, satisfaction with the outcome, psychosocial wellbeing, physical wellbeing and sexual wellbeing) were considered [18].

SPSS software version 27.0 (IBM Corp., Armonk, NY, USA) was used for the simple descriptive statistics, accounting for the patient sociodemographic and clinical characteristics as well as the complications and capsular contracture grade.

The BREAST-Q scores for each patient were converted from the survey scores (1 to 5) to a continuous range from 0 to 100 using QScore Scoring Software. A higher score indicated grater satisfaction or a better HRQOL. To verify the normal distribution of the continuous variables, we used the Shapiro–Wilk test; we then analysed the BREAST-Q scores and expert scores as the continuous variables with a Student’s *t*-test. The discrete variables were analysed with the χ^2^ test. *p*-Values less than 0.05 were considered to be statistically significant.

## 3. Results

We enrolled 84 patients who underwent an SRM with the FNG technique and a prepectoral tissue expander reconstruction implanted with specific covering devices (TiLoop^®^ Bra, PFM medical, Cologne, Germany) between January 2018 and January 2021 in our centre and divided them into two groups using a simple randomisation list. In the first group, 42 patients underwent immediate symmetrisation; in the second group, 42 patients underwent delayed symmetrisation (performed after a median of 9 months).

The characteristics of the study population are collated in Table 1. In the immediate group, the median age was 55.5 years and the average BMI was 24.9; in the delayed group, the median age was 55.8 years and the average BMI was 25.3.

The histology most represented was, in both groups, an invasive ductal carcinoma, with 29 cases (69%) in the immediate symmetrisation group and 28 cases (66.6%) in the delayed symmetrisation group.

In the immediate symmetrisation group, we performed an axillary resection on 10 patients (23.8%) with a macrometastasis at the SNL examination with an OSNA, on 4 after a neoadjuvant CHT and on 32 after sentinel lymph node biopsies (76.2%) (Table 2).

In the delayed symmetrisation group, we performed an axillary resection on 11 patients (26.2%) with a macrometastasis at the SNL examination with an OSNA, on 4 after a neoadjuvant CHT and on 26 after sentinel lymph node biopsies (61.9%) (Table 2). 

The median follow-up time after surgery was 22 months (from 1 to 4 years). The postoperative morbidity is shown in Table 2. Complications requiring a second operation occurred in seven cases: in the immediate symmetrisation group, we reported two wound dehiscence cases (4.8%)—one on the mastectomy side and one on the symmetrisation side—as well as one seroma (2.4%) and one case of skin-nipple necrosis (2.4%), both on the mastectomy side; in the delayed symmetrisation group, we reported one infection (2.4%), one seroma (2.4%) and one case of skin-nipple necrosis (2.4%). We had to remove the tissue expander in three cases because of implant exposure; one in the immediate symmetrisation group and two in the delayed group. In the case of the removal of the prepectoral tissue expander, in two cases a salvage surgery was performed with a submuscular replacement of the tissue expander with the selective denervation of the pectoralis major muscle [5,6] and in one case the tissue expander was removed and a surgical revision was made supplemented with an antibiotate pulse lavage of the pocket surface and a new definitive implant placement [19]. 

Regarding disease recurrence, we reported one case of locoregional cancer recurrence (2.4%) in the delayed symmetrisation group and one case of systemic recurrence (2.4%) in each group. No statistical difference was found between the two groups. The safety and oncological outcomes are reported in Table 3.

As shown in Table 4, we reported two cases (4.8%) in each group significative of capsular contractures (Baker III–IV grade) and in these cases we corrected this issue during the surgical procedure of the definitive implant. We observed a rippling in five cases (12%) in both groups 12 months after the primary surgery. Expander rippling was documented in five breasts (11.9%) in the immediate symmetrisation group and four breasts (9.5%) in the delayed symmetrisation group 12 months after the primary surgery. 

### Measure of the HRQOL and Aesthetic Outcomes

All the patients answered the five domains of the survey. The results are reported, divided for the two different groups, in Table 5. The survey was administered during a follow-up visit 1 year after surgery. The patients scored a high level of satisfaction about the outcomes within each group.

The scores in all the domains were higher in the immediate symmetrisation group, but only the satisfaction with the breasts score had a statistically higher result than the delayed symmetrisation group (*p* < 0.05).

## 4. Discussion

Although the study of breast cancer and its surgical treatment have paved the way for numerous discoveries in the field of oncology [20], there are still technical innovations in both the demolition and reconstructive fields [21,22].

Breast reconstruction during oncological surgery is, today, a recommended practice that provides optimal aesthetic satisfaction to patients and surgeons [13,17]. In an era where continuous innovations such as 3D printing can aid surgical planning [23,24], the search for new materials can radically change surgical tactics. The introduction of biological and synthetic devices aimed at providing an additional layer between the prosthesis and subcutaneous tissue has contributed to prepectoral reconstructions as a predominant role among the reconstructive techniques, reducing the complication rate and increasing the possibility of refining the shape of the breast with fat grafting [25,26,27]. Recent studies in the literature report a small complication rate for this technique with the advantage of a more natural aesthetic result compared with submuscular implants [28,29,30,31,32,33,34].

In this context, SRMs with an FNG for an immediate breast reconstruction are nowadays a preferred surgical strategy for selected patients [7], allowing both a safe oncological clearance and an improved cosmesis [35,36].

The prepectoral approach requires the placement of the tissue expander and the reconstruction to occur in two stages in a few cases when the vascularisation of the skin is not optimal and patients have risk factors such as diabetes, a history of smoking, obesity and a previous RT treatment [14,37].

In the last decade, the need to achieve increasingly satisfactory aesthetic outcomes has led breast surgeons to consider the treatment of the opposite breast as an important aspect of postmastectomy breast reconstructions [38,39,40].

This study aimed to demonstrate the improved outcomes that can be derived from the immediate symmetrisation of the healthy breast during an oncoplastic procedure. Currently, there are no indications of this type of surgical strategy in the literature [11]; however, in our experience, it appeared to us that we were able to guarantee patients a better aesthetic aspect due to the symmetry of the two breasts, especially after a procedure such as an SRM where the asymmetry in quite evident.

Giordano et al. [10] demonstrated that performing immediate symmetrisation at the time of a breast reconstruction was a reasonable and safe option in autologous latissimus dorsii breast reconstructions.

We analysed the satisfaction concerning the cosmetic and functional aspects of patients undergoing a unilateral SRM with an FNG and a prepectoral tissue expander reconstruction through a comparison between the results of patients subjected to immediate symmetrisation and the ones who were candidates for delayed symmetrisation. 

We did not find significant differences in the analysis of the clinical outcomes between the two groups in the study or between these populations and the ones reported in the literature [19,41,42,43]. In two cases, a reintervention was required for implant exposure: one in the symmetrisation group and one in the immediate group.

We also reported an acceptable number of patients with aesthetic complications that required a second surgery; in the majority of cases, a lipofilling with a small quantity of fat grafting was sufficient to correct them [27,44,45]. A high-grade capsular contracture was reported only in 4.8% of cases in each group, according to the literature [46].

In the immediate symmetrisation group, compared with the patients with an SRM and an FNG without symmetrisation, there was a higher subjective satisfaction rate as it improved the aesthetic results by reducing negative self-perception.

## 5. Conclusions

This is, to the best of our knowledge, the first study evaluating immediate symmetrisation during a subcutaneous reconstruction for demolitive surgery. Our findings suggested that immediate symmetrisation was a possible, safe and highly tolerable technique of reconstruction in terms of the aesthetic outcome and the quality of life of the patients. Moreover, immediate symmetrisation did not delay the adjuvant oncological treatments compared with the choice of symmetrisation at the second time of reconstruction.

Furthermore, the consolidated use of covering devices in prepectoral reconstructions in selected patients confirmed how this technique could be applied with a low rate of complications.

This technique, providing patients an aesthetic result in terms of immediate symmetry, allowed us to better manage the waiting times for the second reconstructive surgery whilst still providing an excellent result, even if it was not definitive. Moreover, at the time of the definitive reconstruction, it was possible to evaluate the natural ageing process of the symmetrised breast in order to accordingly adjust the definitive implant of the reconstructed breast. In conclusion, even if not indicated in the literature, it seems to us that the choice of immediate symmetrisation is a viable choice to provide immediate better satisfaction and quality of life for patients and does not excessively impact, from a surgical point of view, on the second time of the reconstruction. 

## Figures and Tables

**Table 1:** Characteristics of the study population.

Characteristics of the Study Population		Immediate Sy.		Delayed Sy.	
		No. of Cases	%	No. of Cases	%
Patients		42		42	
Age		55.5		55.8	
BMI		24.9		25.3	
Histology	Invasive ductal carcinoma	29	69%	28	66.6%
	DCIS	5	11.9%	6	14.2%
	Invasive lobular carcinoma	3	7.2%	4	9.6%
	Invasive ductal carcinoma + DCIS	5	11.9%	4	9.6%
Tumour size	pT1a	1	2.4%	2	4.8%
	pT1b	10	24%	11	26.2%
	pT1c	19	45%	17	40.2%
	pT2	11	26.2%	10	24%
	pT3	1	2.4%	2	4.8%
Pre-BRA	5	7	16.6%	8	19%
	6	14	33.4%	16	38%
	7	12	28.6%	10	24%
	8	9	21.4%	8	19%

**Table 2:** Characteristics of the study population.

Characteristics of the Study Population	Immediate Sy.		Delayed Sy.	
	No. of Cases	%	No. of Cases	%
Axillary dissection (following neoadjuvant CHT)	4	9.50%	4	9.50%
Axillary dissection (without neoadjuvant CHT)	6	14.30%	7	16.70%
Sentinel lymph node biopsy	32	76.20%	31	73.90%

**Table 3:** Safety and oncological outcomes.

Safety and Oncological Outcomes.		Immediate Sy.				Delayed Sy.	
		No. of Cases	%			No. of Cases	%
Tumour Recurrence							
	Locoregional	0	0%			1	2.4%
	Systemic	1	2.4%			1	2.4%
	No recurrence	41	97.6%			40	95.2%
				Symmetrisation side			
Complications	Skin-nipple necrosis	1	2.4%	0	0%	0	0%
	Infection	0	0%	0	0%	1	2.4%
	Wound dehiscence	1	2.4%	1	2.4%	1	2.4%
	Seroma	1	2.4%	0	0%	1	2.4%
Implant Loss		1	2.4%			2	4.8%

**Table 4:** Aesthetic complications.

Aesthetic Complications.		Immediate Sy.		Delayed Sy.	
		No. of Cases	%	No. of Cases	%
Capsular Contracture					
	Grade I	35	83.3%	29	69.0%
	Grade II	5	11.9%	10	23.8%
	Grade III	2	4.8%	1	2.4%
	Grade IV	0		1	2.4%
Rippling		5	11.9%	4	9.5%
Complication Requiring Reoperation		7	16.6%	6	14.3%

**Table 5:** BREAST-Q results.

BREAST-Q	Delayed Sy.	Immediate Sy.	*p*-Value
Satisfaction: breasts	73 ± 10	78 ± 11.9	0.04 *
Psychosocial wellness	76.6 ± 12	79.2 ± 14.2	0.36
Sexual wellness	60.7 ± 12.9	65.3 ± 14.7	0.13
Physical impact	56.5 ± 13.2	58.8 ± 11.8	0.40
Satisfaction with outcome	73 ± 12.1	75 ± 10.7	0.42

* Statistically significant (*p* < 0.05).

## Data Availability

The data presented in this study are available on request from the corresponding author.
